# Comparative genomics of *Klebsiella michiganensis* BD177 and related members of *Klebsiella sp.* reveal the symbiotic relationship with *Bactrocera dorsalis*

**DOI:** 10.1186/s12863-020-00945-0

**Published:** 2020-12-18

**Authors:** Zhaohui Cai, Qiongyu Guo, Zhichao Yao, Wenping Zheng, Junfei Xie, Shuai Bai, Hongyu Zhang

**Affiliations:** State Key Laboratory of Agricultural Microbiology, China–Australia Joint Research Centre for Horticultural and Urban Pests, Institute of Urban and Horticultural Entomology, College of Plant Science and Technology, Huazhong Agricultural University, Wuhan, People’s Republic of China

**Keywords:** Sterile insect technique, Gut microbiota, Probiotics, Phylogenomics classification

## Abstract

**Background:**

*Bactrocera dorsalis* is a destructive polyphagous and highly invasive insect pest of tropical and subtropical species of fruit and vegetable crops. The sterile insect technique (SIT) has been used for decades to control insect pests of agricultural, veterinary, and human health importance. Irradiation of pupae in SIT can reduce the ecological fitness of the sterile insects. Our previous study has shown that a gut bacterial strain BD177 that could restore ecological fitness by promoting host food intake and metabolic activities.

**Results:**

Using long-read sequence technologies, we assembled the complete genome of *K. michiganensis* BD177 strain. The complete genome of *K. michiganensis* BD177 comprises one circular chromosome and four plasmids with a GC content of 55.03%. The pan-genome analysis was performed on 119 genomes (strain BD177 genome and 118 out of 128 published *Klebsiella sp.* genomes since ten were discarded). The pan-genome includes a total of 49305 gene clusters, a small number of 858 core genes, and a high number of accessory (10566) genes. Pan-genome and average nucleotide identity (ANI) analysis showed that BD177 is more similar to the type strain *K. michiganensis* DSM2544, while away from the type strain *K. oxytoca* ATCC13182. Comparative genome analysis with 21 *K. oxytoca* and 12 *K. michiganensis* strains, identified 213 unique genes, several of them related to amino acid metabolism, metabolism of cofactors and vitamins, and xenobiotics biodegradation and metabolism in BD177 genome.

**Conclusions:**

Phylogenomics analysis reclassified strain BD177 as a member of the species *K. michiganensis.* Comparative genome analysis suggested that *K. michiganensis* BD177 has the strain-specific ability to provide three essential amino acids (phenylalanine, tryptophan and methionine) and two vitamins B (folate and riboflavin) to *B. dorsalis.* The clear classification status of BD177 strain and identification of unique genetic characteristics may contribute to expanding our understanding of the symbiotic relationship of gut microbiota and *B. dorsalis*.

**Supplementary Information:**

The online version contains supplementary material available at 10.1186/s12863-020-00945-0.

## Background

*Bactrocera dorsalis* (Hendel) (Diptera: Tephritidae) is a destructive polyphagous and highly invasive insect pest of tropical and subtropical species of fruit and vegetable crops. While insecticides have been used to control this pest, insecticide resistance, and environmental pollution by chemical pesticides have been severely limiting this type of control [1]. Moreover, *B. dorsalis* has a powerful biological invasion ability. It is its invasion, spread, and establishment in sub-Saharan Africa and has caused about $2 billion in economic losses in the horticultural export markets of Africa [2]. The current movement of *B. dorsalis* into Central China, without any apparent intense selective pressure, must pose a deep concern for other temperate regions of the world, especially Europe and North America [3].

The sterile insect technique (SIT) has been used for decades to control insect pests of agricultural, veterinary and human health importance [4, 5]. Compared with insecticide control strategies, SIT has several attractive features, including species specificity and environment friendliness. Ionizing irradiation was used to sterilize insects, and these insects were subsequently handled, transported, and released in the field, ideally only males [6]. Thus, SIT can be an alternate strategy for the management of *B. dorsalis*. Previously, SIT has been used to control pest fruit fly species, including *Ceratitis capitata* [7], *B. tryoni* [8], *B. cucurbitae* [9] and *B. dorsalis* [10]. However, SIT may have some limitations related to the ecological fitness of sterile male adult flies due to domestication, mass-rearing, irradiation, and handling [11]. These procedures also impact the tephritid gut microbiome, with detrimental effects on physiology, behavior, and fitness [12, 13]. Thus, the deleterious impact on the ecological fitness of the released insects has been one of the most considerable issues of SIT applications [11, 14].

Gut microbiota is strongly connected with the biology of the host and contributes to its health [15]. Gut microbiota affects insects in several ways, such as aiding food digestion and detoxification [16], providing essential nutrients [17], and protecting against infectious pathogens [18]. Much recent research of Tephritids suggests that gut microbiota reduced larval development time [19, 20], increased pupal weight [21], larger males [22], improved male performance [13, 23], increased female fecundity [24], increased longevity [23, 25] and increased chilling resistance [26]. Mass rearing and irradiation processes affect the gut microbial community structure in the Tephritids [13, 23, 27, 28]. Compared to wild flies, the abundance and diversity of the major gut microbiota community Enterobacteriaceae in mass-reared irradiated flies are reduced, and the abundance of the minor members (e.g., Pseudomonas or Bacillaceae) increased [13, 23]. This disturbance of gut microbial homeostasis may be causally related to the competitiveness disadvantaged of sterile males. The above researches show that the manipulation of gut microbiota has great potential and can be introduced to SIT facilities to improve the efficiency of pest control. Gut bacteria can be used as probiotics to prevent pathogens, promote larval growth, and male performance during all the production stages of sterile male fly, from the egg to the released fly.

Gut symbiotic bacteria community of *B. dorsalis* has been investigated [23, 27, 29]. Enterobacteriaceae were the predominant family of different *B. dorsalis* populations and different developmental stages from laboratory-reared and field-collected samples [27, 29]. Our previous study found that irradiation causes a significant decrease in Enterobacteriaceae abundance of the sterile male fly [23]. We succeed in isolating a gut bacterial strain BD177 (a member of the Enterobacteriaceae family) that can improve the mating performance, flight capacity, and longevity of sterile males by promoting host food intake and metabolic activities [23]. However, the probiotic mechanism remains to be further investigated. Therefore, the genomic characteristics of BD177 may contribute to an understanding of the symbiont-host interaction and its relation to *B. dorsalis* fitness. The here presented study aims to elucidate the genomic basis of strain BD177 its beneficial impacts on the sterile males of *B. dorsalis.* An insight into strain BD177 genome feature helps us make better use of the probiotics or manipulation of the gut microbiota as an important strategy to improve the production of high performing *B. dorsalis* in SIT programs.

## Results

### Assembly description and genome information of *K. michiganensis* BD177

The genome of *K****.*** *michiganensis* BD177 was sequenced using the Illumina HiSeq and Pacbio technology. Raw reads (~ 1.3 Gbp) were processed to remove SMRT bell adapters, short and low-quality reads (< 80% accuracy) using SMRT Analysis version 2.3 (https://www.pacb.com/products-and-services/analytical-software/whole-genome-sequencing/). A total of 155,828 filtered reads (average length, 8.9 Kb) were used for de novo assembly using Celera Assembler [30], with self-correction of the PacBio reads. A total of 1091 Mb paired-end Illumina reads screened from a 500-bp genomic library were mapped using SOAP [31] to the bacterial plasmid database for tracing the presence of any plasmid and filling the gaps. The de novo assembly resulted in five contigs, representing the *K****.*** *michiganensis* BD177 complete genome of 6,812,698 bp with GC content 55.03% in a single chromosome and four plasmids. The annotated genome contains 6714 genes, 25rRNA genes, and 86 tRNA genes (Table 1).

**Table 1:** Genome features of isolated strain BD177

Features	BD177
Genome Size (bp)	6,812,698
Length of Chromosome (bp)	6,150,416
Numberof plasmids	4
Length of Plasmid1 (bp)	281,962
Length of Plasmid2 (bp)	191,962
Length of Plasmid3 (bp)	164,510
Length of Plasmid4 (bp)	23,848
GC content (%)	55.03%
No. of 5 s rRNA	9
No. of 16 s rRNA	8
No. of 23 s rRNA	8
No. of tRNA	86
Genes	6714

### 16S rRNA phylogenetic analysis and phenotypic of isolated strain BD177

Maximum parsimony based and maximum likelihood phylogenetic trees using the sequence that encodes for the 16S rRNA gene showed that the closest species to BD177 is *K. michiganensis* and *K. oxytica* (Additional file 1: Fig. S1, Additional file 2: Table S1). Evolutionary divergence distance of 16S rRNA gene sequence between BD177 and *K. michiganensis* E718 was minimum value (BD177 vs *K. michiganensis* E718: 0.0079) among all sequences. Biochemical tests were performed on BD177 by API20E to confirm its position in the *K. oxytoca* and *K. michiganensis*. Biochemical characteristics of *K. oxytoca* ATCC13182(T)(=NCTC13727) and *K. michiganensis* W14(T)(= DSM2544) were obtained from BacDive Webservices [32]. Compared with *K. oxytoca* ATCC13182(T) and *K. michiganensis* W14(T), BD177 is the same with W14 in the β-galactosidase enzyme positive and urease negative, same with ATCC13182(T) in arginine dihydrolase positive. However, BD177 does not have the utilization ability of citrate as only carbon source, different from both type strains (Table 2).

**Table 2:** Phenotypic profiles of the isolated strain BD177

Characteristics	BD177	***Klebsiella michiganensis*** W14(T)	***Klebsiella oxytoca*** ATCC13182(T)
**ONPG**	**+**	**+**	**–**
**ADH**	**+**	**–**	**+**
**LDC**	**+**	**+**	**+**
**ODC**	**–**	**–**	**–**
**CIT**	**–**	**+**	**+**
**H2S**	**–**	**–**	**–**
**URE**	**–**	**–**	**+**
**TDA**	**–**	**–**	**–**
**IND**	**+**	**+**	**+**
**VP**	**+**	**+**	**+**
**GEL**	**–**	**–**	**–**
**GLU**	**+**	**+**	**+**
**MAN**	**+**	**+**	**+**
**INO**	**+**	**+**	**+**
**SOR**	**+**	**+**	**+**
**RHA**	**+**	**+**	**+**
**SAC**	**+**	**+**	**+**
**MEL**	**+**	**+**	**+**
**AMY**	**+**	**+**	**+**
**ARA**	**+**	**+**	**+**
**OX**	**–**	**–**	**–**

*ONPG* β-galactosidase enzyme; *ADH* arginine dihydrolase; *LDC* lysine decarboxylase; *ODC* ornithine decarboxylase; *CIT* utilization of citrate as only carbon source; *H2S* production of hydrogen sulfide; *URE* urease; *TDA* tryptophan deaminase; *IND* tryptophanase; *VP* acetoin produced from glucose; *GEL* gelatinase; *GLU* fermentation of glucose; *MAN* fermentation of mannose; *INO* fermentation of inositol; *SOR* fermentation of sorbitol; *RHA* fermentation of rhamnose; *SAC* fermentation of sucrose; *MEL* fermentation of melibiose; *AMY* fermentation of amygdalin; *ARA* fermentation of arabinose; *OX* cytochrome-c oxidase; Result: +, positive; −, negative

### Pan-genome analysis

**Fig. 1:**
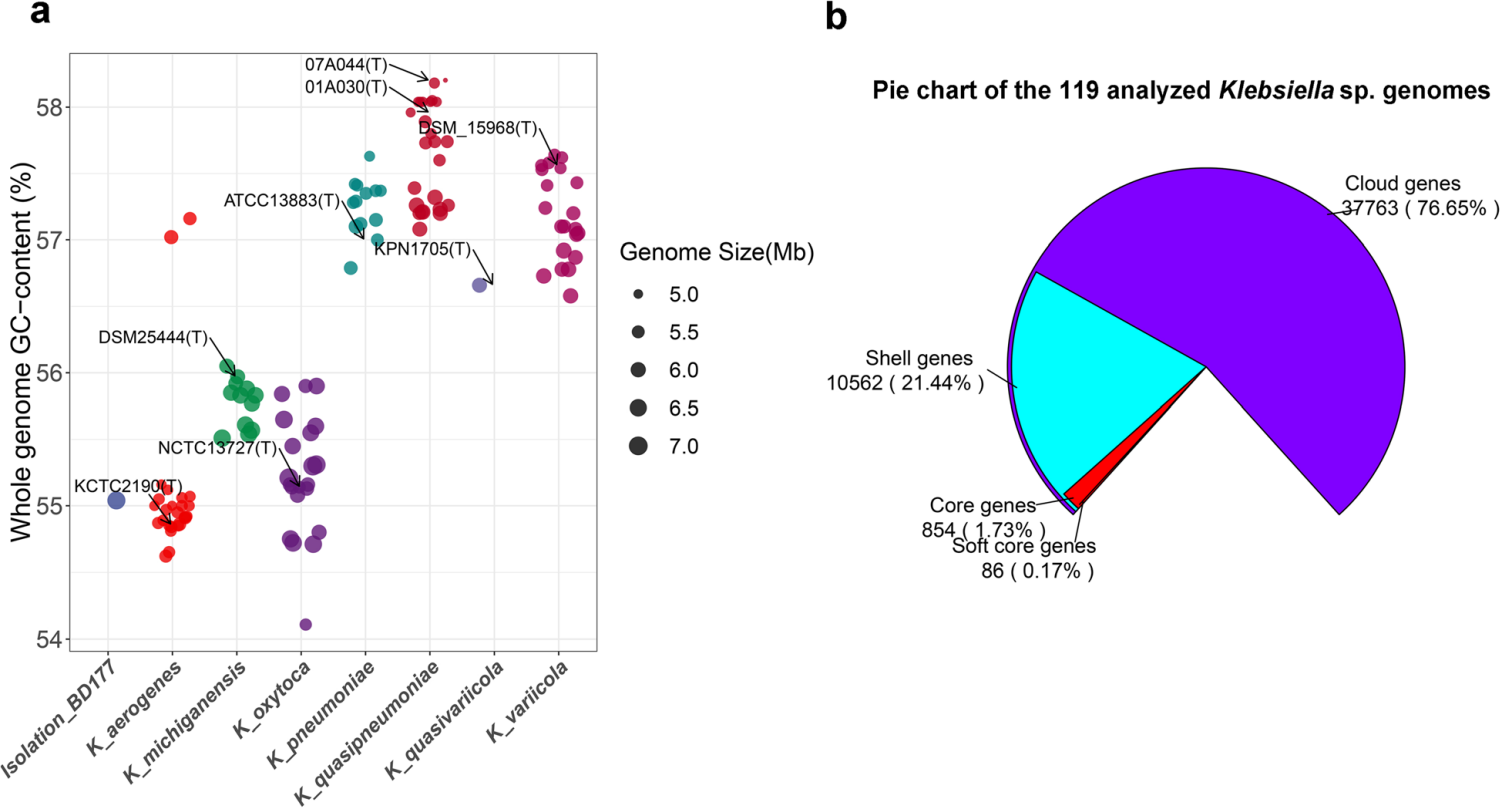
Pan-genome analysis of 119 genomes in this study. Genomes are grouped according to their species annotation in the NCBI database (except for isolate *K. michiganensis* BD177). **a** GC content of all genomes is grouped according to their species annotation in the NCBI database. **b** pie chart of the 119 analyzed *Klebsiella* sp. genomes. The pan-genome pie chart showing gene content visualized with the use of Roary software

In this study, we considered the 128 publicly available genome assemblies for the *Klebsiella* sp*.* (Additional file 3: Table S2). Of these genomes, 26 were originally annotated as *K. aerogenes*, 13 were *K. michiganensis*, 27 were *K. oxytoca*, and 15 were *K. pneumoniae*. 25 were *K. quasipneumoniae*, 1 was *K. quasivariicola*, 21 were *K. variicola.* The type strain genome of each species is included in these genomes. These genome assemblies were passed strict quality control (N75 values of > 10,000 bp, < 500 undetermined bases per 100,000 bases, > 90% completeness and < 5% contamination) by quast and checkm (Additional file 4: Table S3). This resulted in a total of 118 *Klebsiella* sp*.* strains studied, where ten low-quality genome assemblies were discarded. The GC contents of the species *K. michiganensis, K. oxytoca, K. pneumoniae, K. variicola,* and *K. quasipneumoniae* showed low intraspecies variation, with respective average values of 55.78, 55.22, 57.25, 57.18 and 57.62% (Fig. 1a), whereas larger interspecies diversity was observed. In contrast, *K. aerogenes* genomes can be divided into two groups, a large group showing a GC content in the range of that of *Klebsiella aerogenes* (54.62 to 55.16%) and a small group of two genomes (57.02 and 57.16%) showing a much greater GC content, similar to that of the *K. pneumoniae, K.variicola* and *K. quasipneumoniae* genomes (respective average values about 57%). Based on GC content of the genome*, K. pneumoniae, K.variicola, K. quasivariicola*, and *K. quasipneumoniae* were considered as a high GC group content, and *K. michiganensis* and *K. oxytoca* were considered as a low GC content group. The genome sequence of our new isolate, which we designated *K. michiganensis* BD177 with 55.03% GC content (Table 1), shows a similar to that of the low GC content *Klebsiella* sp*.* group (Fig. 1a).

**Fig. 2:**
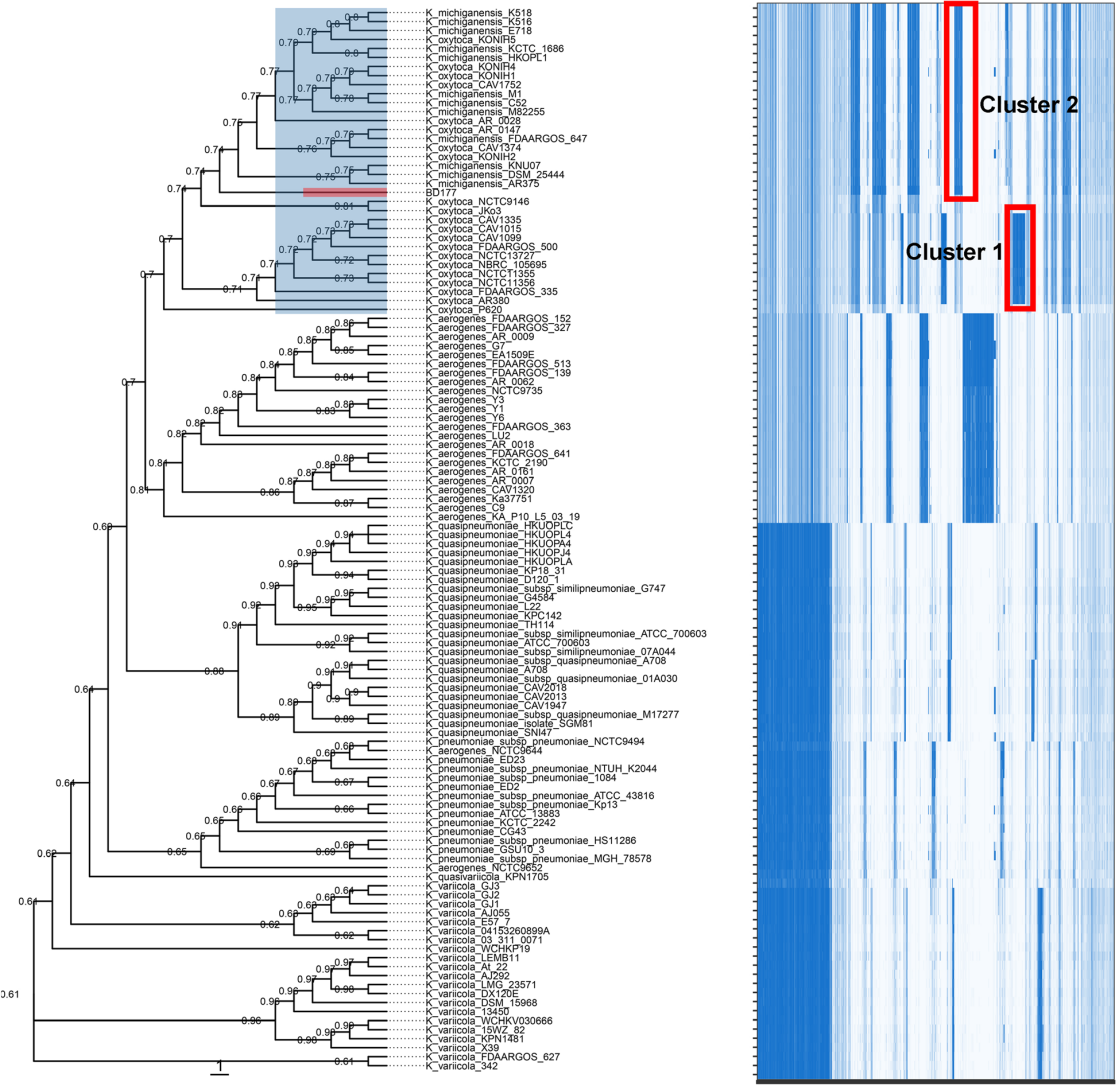
Phylogenomics analysis of BD177 based on pan-genome. A heatmap was indicating gene presence (dark blue) or absence (light blue) in each of the 119 strains. A phylogeny built based on the presence and absence of core and accessory genes is shown on the left, and the strain names are indicated on the right

The pan-genome shape of the 119 analyzed *Klebsiella sp.* genomes is presented in Fig. 1b. Hard core genes are found in > 99% genomes, soft core genes are found in 95–99% of genomes, shell genes are found in 15–95%, while cloud genes are present in less than 15% of genomes. A total of 49,305 gene clusters were found, 858 of which comprised the core genome (1.74%), 10,566 the accessory genome (21.43%), and 37,795 (76.66%) the cloud genome (Fig. 1b). Comparative genomic analysis evidenced that the 119 *Klebsiella sp.* pangenome can be considered as “open” since nearly 25 new genes are continuously added for each additional genome considered (Additional file 5: Fig. S2). To study the genetic relatedness of the genomic assemblies, we constructed a phylogenetic tree of the 119 *Klebsiella sp.* strains by using the presence and absence of core and accessory genes from pan-genome analysis (Fig. 2). The tree structure reveals six separate clades within 119 analyzed *Klebsiella sp.* genomes (Fig. 2). From this phylogenetic tree, type strain genomes originally annotated *K. aerogenes, K. michiganensis*, *K. oxytoca, K. pneumoniae, K.variicola,* and *K. quasipneumoniae* in the NCBI database were divided into six different clusters. Some non-type strain genomes originally annotated *as K. oxytoca* in the NCBI database are clustered in type strain *K. michiganensis* DSM25444 clade. The *K. oxytoca* group, including type strain *K. oxytoca* NCTC13727, have the unique gene cluster 1 (Fig. 2). *K. michiganensis group*, including type strain *K. michiganensis* DSM25444, has the unique cluster 2 (Fig. 2). Genes cluster 1 and cluster 2 based on unique presence genes from the pan-genome analysis can distinguish between non-type strain *K. michiganensis* and *K. oxytoca* (Fig. 2). However, our new isolated BD177 is clustered in type strain *K. michiganensis* clade (Fig. 2)*.*

**Fig. 3:**
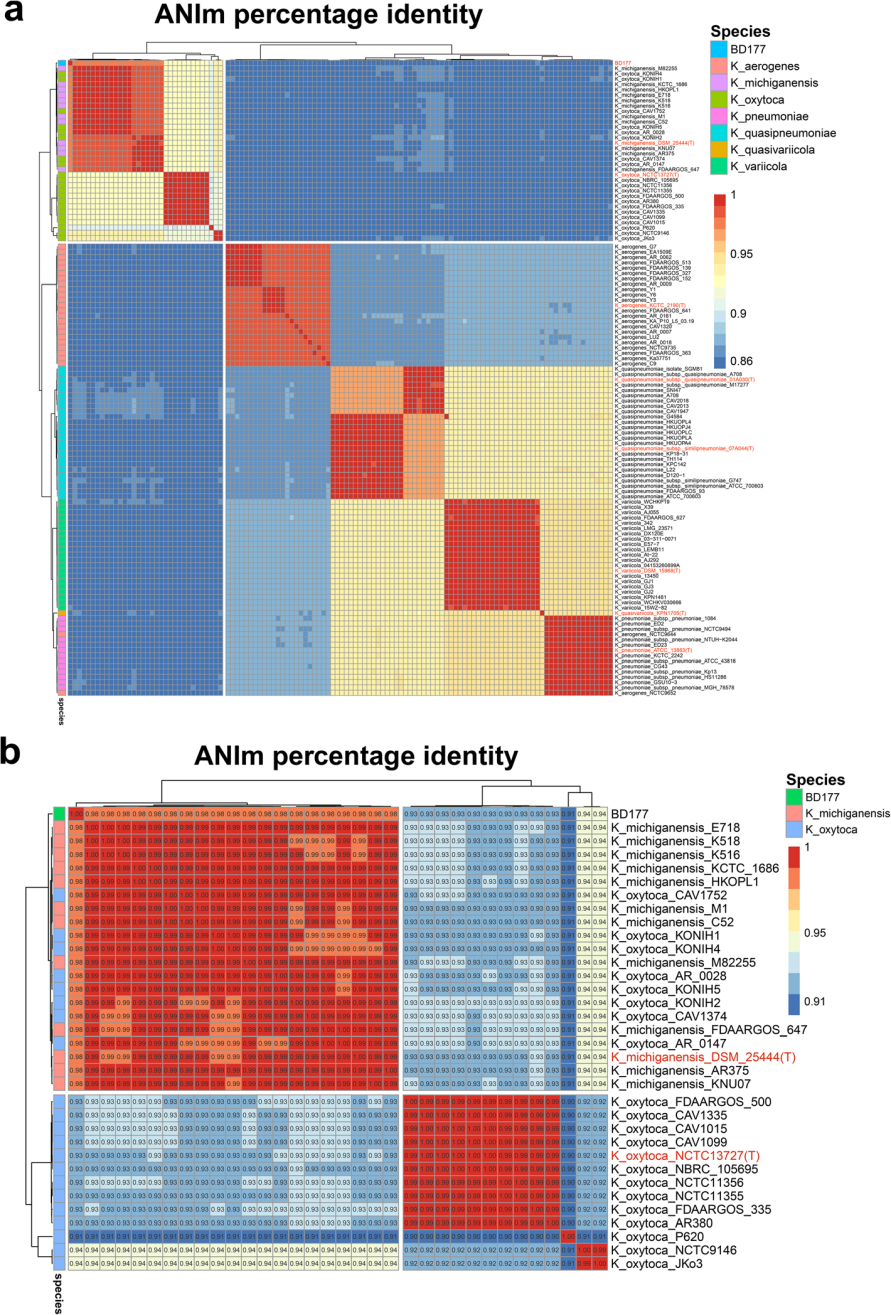
Pairwise ANIm values for genomes. **a** Heatmap and hierarchal clustering of average nucleotide identity (ANI) values between BD177 and 118 *Klebsiella* sp. strains. See supplement for a table with ANI values (Additional file 7: Table S4). **b** Pairwise ANIm values for genomes. Heatmap and hierarchal clustering of average nucleotide identity (ANI) scores between BD177, 21 *K. oxytoca* and 12 *K. michiganensis* strains

In the current era of high-throughput sequencing with easy access to bacterial genomes, average nucleotide identity (ANI) of genome-wide comparison has been recommended as a standard method to improve the accuracy of taxonomic identification of prokaryotic genomes [33]. Figure 3a shows the ANIm (ANI calculated by using a MUMmer3 [34] implementation) between all 119 *Klebsiella sp.* genomes. The distance metrics support the grouping of the genomes in the six clades defined by the phylogenetic tree in Fig. 2. For species delimitation, Ciufo et al. [35] advise the use of an ANI cutoff of 96% to define species boundaries. When comparing genomes belonging to different *Klebsiella* species, we observed ANI values of ≤94.8% (Fig. 3b and Additional file 6: Table S4), indicating that each clade consists of one species(expect for *K.oxytoca* P620, *K. oxytoca* NCTC9146, and *K. oxytoca* JKo3) that are distinct from the other clades (Fig. 3b). The average nucleotide identity (ANI) value between BD177 and type strain *K. michiganensis* DSM25444 was 98% more than the cutoff of 96% (Fig. 3b), while between BD177 and type strain *K. oxytoca* NCTC13727 was 93%.

### Genetic potential of BD177 for a symbiotic relationship

**Fig. 4:**
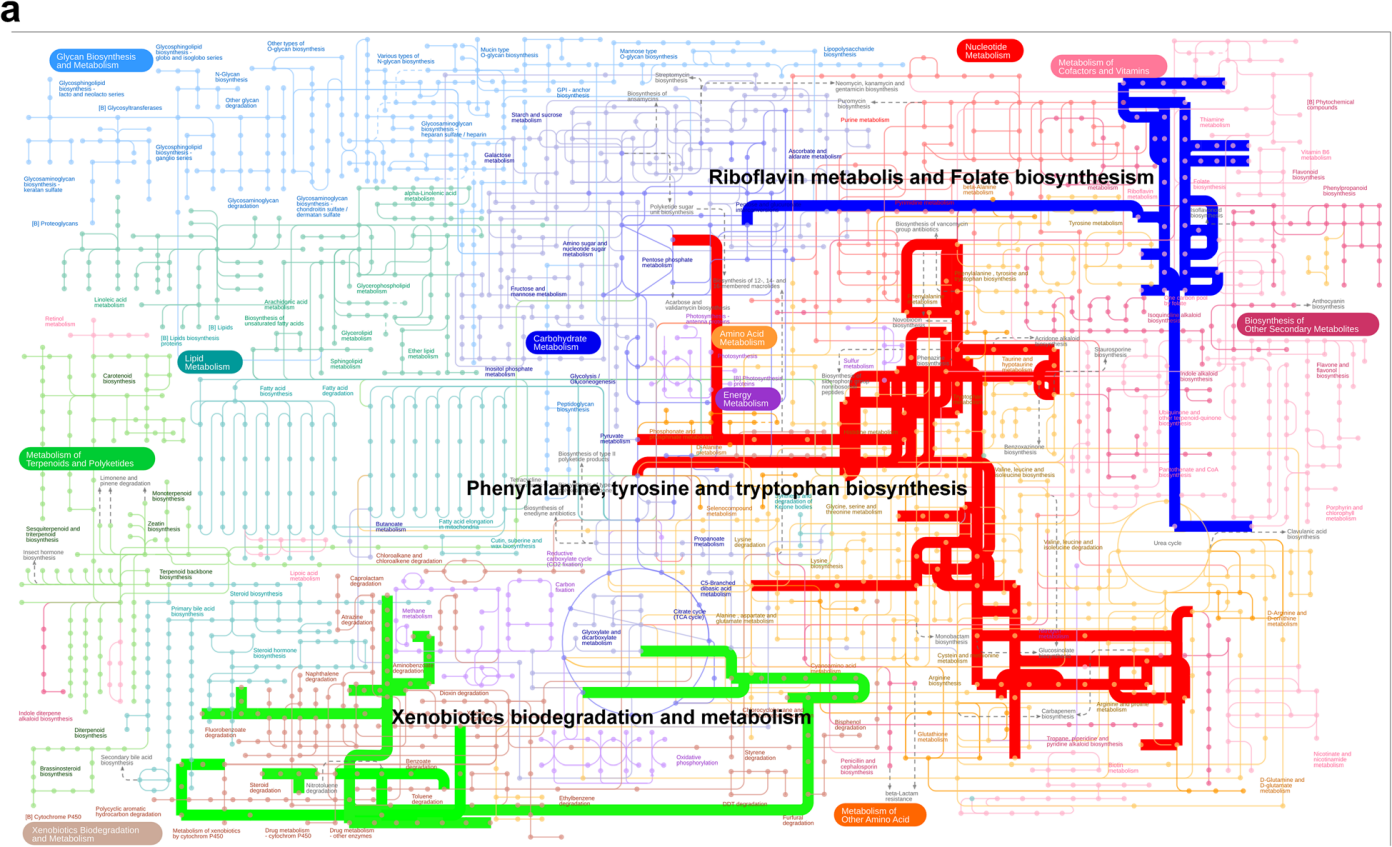
Metabolic pathways of BD177 strain. The pathways were generated using the iPath (ver. 3) module and are based on KEGG Orthology numbers of orthogroups identified from the pan-genome analysis of 34 genomes. Metabolic pathways identified from unique orthogroups of BD177 are depicted in blue, red and green, respectively

Based on the pan-genome analysis of 119 *Klebsiella sp.* strains, we focused on the genomic relatedness of *K. michiganensis* BD177, 21 *K. oxytoca,* and *12 K. michiganensis* strains. In total, 201,774 genes were present in their genomes, with an average number of 5934 genes per genome. These genes were clustered into 9643 orthogroups by OrthoFinder [36], where an orthogroup is defined as the group of genes descended from a single gene in the most recent common ancestor of a group of species. Of these orthogroups, 3833 were identified as core orthogroups, and 3351 were identified as single-copy orthogroups (Additional file 7: Table S5). In *K. michiganensis* BD177, 213 strain-specific orthogroups were identified. Mapping of these orthogroups to the eggNOG database (v4.5) [37] revealed a unique in the predicted functional capacity (Additional file 8: Table S6). The metabolic features of *K. michiganensis* BD177 were also investigated by cumulatively mapping the unique functional KEGG genes of the *K. michiganensis* BD177 to the KEGG pathways (Fig. 4). The KEGG pathway analysis showed that BD177 harbored complete phenylalanine, tyrosine, and tryptophan biosynthesis pathways (KEGG genes K04518, K04093, K11646), and Cysteine and methionine metabolism pathways (KEGG genes K13060). The KEGG pathway analysis also showed that the BD177 strains harbored Metabolism of cofactors and vitamins pathway for riboflavin metabolism (KEGG genes K19286) and folate biosynthesis (KEGG genes K06920). In addition, BD177 strains harbored genes of the xenobiotics biodegradation and metabolism pathway (KEGG genes K01061 and K05797) for chlorocyclohexane and chlorobenzene degradation, fluorobenzoate degradation, and toluene degradation.

## Discussion

*Klebsiella* sp*.* strains are among the dominant symbiotic bacteria in the gut of Tephritidae, with the ability to play diverse roles [11]. In this study, we describe the complete genome sequence of *K. michichanensis* BD177, isolated from wild adult *B. dorsalis*. We found that the taxonomic status of the *B. dorsalis* gut bacterial strain BD177 could not be identified only based on the 16S rRNA gene phylogenetic tree and biochemical analysis of the strain. Hence, we used the Illumina HiSeq and Pacbio technology to assemble a complete 6.8 Mbp length genome of *K. michiganensis* BD177. Using the genome-wide information, we identified the taxonomic status and strain-specific genome features of the BD177 strain to explore the symbiotic relationship with *B. dorsalis.*

The phylogenetic analysis of the 16S rRNA gene shows that the closest species to BD177 is *K. oxytoca* and *K. michiganensis* (Fig. S1). Bootstrap values were more than 70%, which corresponds to a probability of ≥95% that the corresponding clade is real [38]. In addition, the biochemical indicators of the BD177 strain are not the same as the type strain of *K. oxytoca* and *K. michiganensis*(Table 2). Thus, the species status of the strain BD177 is still unclear. The 16S rRNA gene is often used as a putative marker for species circumscription, but the conservative nature of the gene did not show enough resolution on such a taxonomic scale [33]. Phenotypic properties can be unstable at times, and expression can be dependent upon changes in environmental conditions, e.g., growth substrate, temperature, and pH levels [39]. Furthermore, biochemical properties do not accurately reflect the entire extent of the genomic complexity of a given species [39].

As whole-genome sequencing has become more widely accessible due to the introduction of cost-effective high-throughput DNA sequencing technology, it is evident that genome sequence similarities have been developed to be a routine taxonomic parameter. We compared the genome feature of BD177 with 118 currently available high-quality genomic assemblies of the *Klebsiella sp.*, which comprises the species *K. aerogenes, K. michiganensis*, *K. oxytoca, K. pneumoniae, K. variicola,* and *K. quasipneumoniae.* Here we clarified the classification status of *K. michiganensis* BD177, compared with the six taxonomic clades on the basis of (i) differences in whole-genome GC content (Fig. 1a), (ii) a phylogenetic tree constructed on the presence and absence of core and accessory genes from pan-genome analysis (Fig. 2), and (iii) pairwise ANI (Fig. 3). All 118 *Klebsiella* sp*.* genomes were divided into a low GC content group (including *K. aerogenes, K. michiganensis* and *K. oxytoca* species) and a high GC content group (including *K. pneumoniae, K. variicola* and *K. quasipneumoniae* species). Strain BD177 with 55.03% GC content belongs to the low GC genome group. The GC content of complex microbial communities seems to be globally and actively influenced by the environment. Similar environments tend to have similar GC-content patterns [40]. A phylogenetic tree constructed on the presence and absence of core and accessory genes confirms the position of strain BD177 within the *Klebsiella* sp. strains. The *K. michiganensis* group, including type strain *K. michiganensis* DSM25444, have the unique gene cluster 2. *K. oxytoca group*, including type strain *K. oxytoca* NCTC13727, has the unique cluster 1. Non-type strains *K. michiganensis* and *K. oxytoca* are distinguishable based on genes cluster 1 and cluster 2 from the pan-genome analysis. Whole-genome sequence data as a basis for taxonomic assignment display greater discriminatory power than 16S rRNA gene sequence analysis alone [41]. In addition, pairwise genome comparison metrics such as average nucleotide identity (ANI) is also used as a reliable method to verify taxonomic identities in prokaryotic genomes, for both complete and draft assemblies [33]. Based on average nucleotide identity (ANI) value with the type strain *K. michiganensis* and *K. oxytoca* similarity, BD177 belongs to *K. michiganensis* species rather than *K. oxytoca* species (Fig. 3b). This result is consistent with the phylogenetic analysis base on the pan-genome. Strain BD177 belongs to *K. michiganensis.*

To explore potential probiotic of *K. michiganensis* BD177, an in-depth comparative genomics analysis of 34 genomes, including 21 *K. oxytoca*, 12 *K. michiganensis* and *K. michiganensis* BD177, was performed by Orthofinder. We found the 213 strain-specific orthogroups of the strain BD177 were identified from a total of 9643 orthogroups in comparative genomics analysis. Predicted functional capacity analysis of these orthogroups showed that these unique orthogroups include metabolic key enzymes of amino acid, vitamins and xenobiotics. Of potential importance to the symbiosis of strain BD177 with the insect, the host is the bacterium’s encoded ability to biosynthesize the phenylalanine, tyrosine, tryptophan, cysteine and methionine. Our previous research also showed that supplementation of *K. michiganensis* BD177 to sterile male *B. dorsalis* improved total free amino acid levels in hemolymph [23]. The obligate primary endosymbionts of many sap-feeding insects provide their hosts with essential amino acids [42–44]. The symbiotic fungi of *Drosophila melanogaster* promote amino acid harvest to rescue the lifespan of undernourished flies [45]. It suggested that *K. michiganensis* BD177 can provide amino acids, especially essential amino acids such as phenylalanine, tryptophan and methionine, to the *B. dorsalis*.

Additionally, *K. michiganensis* BD177 was found to encode the ability to biosynthesize the B vitamins riboflavin (B2) and folate (B9). In humans, gut microbiota can synthesize and supply vitamins B to their hosts, which lack the biosynthetic capacity for most vitamins [46]. Recent studies have implicated the *Drosophila* microbiota in supplying folate [47], riboflavin [48] and thiamine [49]. The riboflavin and folate biosynthesize ability of *K. michiganensis* BD177 suggests these B-vitamins may be of particular importance, especially in adult life stages fed on undernourished nectar and dew [50]. In *D. melanogaster, Acetobacter pomorum* provides thiamine to its host to promote larval development [49]. Folate (B9) biosynthesis of *Wigglesworthia glossinidia* plays a role in *Glossina morsitans* maturation and reproduction [51]. Our previous research also showed that *K. michiganensis* BD177 improved the mating competitiveness and lifespan of sterile male *B. dorsalis* [23]. Additionally, the recent study reported that *K. oxytoca* could affect the foraging decision [52] and mate-selection [53] of *B. dorsalis*. It is suggested that riboflavin and folate synthetic ability of *K. michiganensis* BD177 may contribute to the sexual performance and lifespan of *B. dorsalis*.

Compared with other 33 *Klebsiella sp.* genomes, some strain-specific genes from xenobiotics biodegradation and metabolism pathway were also identified in the *K. michiganensis* BD177 genome. Recent research shows that gut bacteria of insects significantly contribute to resistance against xenobiotics, including phytotoxins and pesticides [54]. *Pseudomonas fulva* can assist with digestion and detoxification of caffeine as alkaloid allelochemical in the coffee berry borer [16]. Gut symbiont *Burkholderia* of *Riptortus pedestrians* has gained the ability to hydrolyze insecticide fenitrothion [55]. Gut symbiont *Citrobacter sp.* can degrade trichlorphon and conferred host insecticide resistance in *B. dorsalis* [56]. Interestingly, gut symbiont *Lactobacillus plantarum* of *D. melanogaster* can significantly enhance the toxicity of insecticide chlorpyrifos [57]. It is hypothesized that *K. michiganensis* BD177 may play a role in insect resistance against pesticides in the wild field. However, the potential mechanisms of resistance or sensitivity of insect to insecticide are unclear.

## Conclusions

Supplement of gut symbiotic bacteria as probiotic to the larval or adult diet are encouraging for their potential application in SIT programs to produce high-quality insects. However, understanding of the bacterial probiotic mechanism is important for the selection and application of different insect species probiotics. Using long-read sequence technologies, we assembled the complete genome of *K. michiganensis* BD177 strain. The comparison of the genome sequence against other *Klebsiella* species showed a percentage of genes that are unique to the BD177 strain, including metabolic key enzymes of amino acid, vitamins, and xenobiotics that could play an important role in the resistance and fitness of *B. dorsalis*. These findings extend our previous work [23] to improve the understanding of the relationship between gut bacteria and *B. dorsalis*. In the future, we can engineer the gut bacteria strain symbionts by strengthening the probiotic genetic elements of bacteria. It will improve the application efficiency of gut microbiota in pest management programs incorporating SIT.

## Methods

### 16S rRNA gene analysis and biochemical characterization

The *Klebsiella michiganensis* BD177 reported in this study was isolated in a previous study from the gut of *B. dorsalis* male adult, collected from the Institute of Urban and Horticultural Pests of Huazhong Agricultural University [23]. The bacterial DNA was extracted with the HiPure Bacterial DNA Kit (Magen) following the protocol for Gram-negative bacteria and used for the amplification of 16S rRNA gene using the primers 27F (5′-GTTTGATCCTGGCTCAG-3′) and 1492R (5′-GGTTACCTTGTTACGACTT-3′) [16]. Subsequently, the ~ 1.4 kb PCR product was purified using a PCR purification kit (Axygen) and was subjected to bidirectional Sanger sequencing. The 16S rRNA gene sequence of strain BD177 was compared with the reference sequences using the “identify” tool of EzBioCloud database for the taxonomic assignment [58]. The similarity of the 16S rRNA gene between strain BD177 to *Klebsiella michiganensis* type strain W14 and *Klebsiella oxytoca* type strain JCM 1665 was 99.25 and 99.15%, respectively. In addition, 16S rRNA gene sequences longer than 1300 nucleotides of type strains *K. oxytoca*, *K. michiganensis, K. pneumonia*, *K. quasipneumoniae*, *K. aerogenes*, *Klebsiella variicola,* and *Pseudomonas aeruginosa* were downloaded from EzBioCoud database [58]. Alignments of the sequences were performed using the MUSCLE software [59]. A neighbor-joining phylogenetic tree based on sequences of the 16S rRNA gene was constructed with the Molecular Evolutionary Genetics Analysis (MEGA X) [60], using the Kimura 2-parameter model with 1000 bootstrap replicates. The maximum parsimony and maximum likelihood trees with the inclusion of outgroup were constructed by MEGA X. Distance matrix of evolutionary divergence between 16 s rRNA gene sequences was estimated by MEGA X. Strain BD177 was biochemically confirmed by using the API 20E system according to the manufacturer’s instructions, which is a biochemical panel for identification and differentiation of members of the family Enterobacteriaceae (bioMerieux Inc., Hazelwood, MO) [61].

### DNA isolation and genome sequencing

*K. michiganensis* BD177 strain was grown on LB, with incubation at 37 °C in aerobic conditions. For DNA extraction, the strain was grown in 8 ml of medium for overnight, followed by pelleting at 6000 g for 10 min, and genomic DNA was obtained using E.Z.N.A.® Bacterial DNA Kit (OMEGA). The bacterial DNA extraction was followed by quantity and quality estimation using a NanoDrop (Thermo Scientific) and also visualization of aliquots onto a 1.2% agarose gel stained with ethidium bromide to verify DNA integrity. Whole-genome sequencing of *Klebsiella michiganensis* BD177 was performed on the PacBio RS II platform and Illumina HiSeq 4000 platform at the Beijing Genomics Institute (BGI, Shenzhen, China). For Illumina sequencing, genomic DNA was sheared randomly to construct three read libraries with lengths 500 bp by a Bioruptor ultrasonicator (Diagenode, Denville, NJ, USA) and physicochemical methods. DNA was sequenced on a HiSeq sequencer (Illumina) with pair-end 125 bp reads. Low-quality trimming and adapter removal for the Illumina reads was performed using Trimmomatic [62], resulting in a total of 1091 Mb clean data. For Pacbio sequencing, the program pbdagcon (https://github.com/PacificBiosciences/pbdagcon) was used for self-correction. Then these reads were filtered using the RS_Subreads protocol (minimum subread length = 1 kb, minimum polymerase read quality = 0.8), resulting in a total of 1394 Mb useable data (total number of subreads = 155,828 reads, mean subread length = 8897 bp, subread N50 = 11,669 bp).

### Genome assembly and annotation of *K. michiganensis* BD177

Draft genomic unitigs, which are uncontested groups of fragments, were assembled using the Celera Assembler [30] against a high quality corrected circular consensus sequence subreads set. To improve the accuracy of the genome sequences, GATK (https://www.broadinstitute.org/gatk/), and SOAP tool packages (SOAP2, SOAPsnp, SOAPindel) were used to make single-base corrections [31]. To trace the presence of any plasmid, the filtered Illumina reads were mapped using SOAP [31] to the bacterial plasmid database (last accessed 5 March 2018) [63].

Gene prediction was performed on the *K. michiganensis* BD177 genome assembly by glimmer3 [64] with Hidden Markov Models. tRNA, rRNA, and sRNAs recognition made use of tRNAscan-SE [65], RNAmmer [66] and the Rfam database [67]. The tandem repeats annotation was obtained using the Tandem Repeat Finder [68], and the minisatellite DNA and microsatellite DNA selected based on the number and length of repeat units. The Genomic Island Suite of Tools (GIST) used for genomics lands analysis [69] with IslandPath-DIOMB, SIGI-HMM, IslandPicker method. Prophage regions were predicted using the PHAge Search Tool (PHAST) webserver [70] and CRISPR identification using CRISPRFinder [71].

Seven databases, which are KEGG (Kyoto Encyclopedia of Genes and Genomes) [72], COG (Clusters of Orthologous Groups) [73], NR (Non-Redundant Protein Database databases) [74], Swiss-Prot [75], and GO (Gene Ontology) [76], TrEMBL [75], EggNOG [37] are used for general function annotation. A whole-genome BLAST search (E-value below 1e− 5, minimal alignment length percentage above 40%) was performed against the above seven databases. Virulence factors and resistance genes were identified based on the core dataset in VFDB (Virulence Factors of Pathogenic Bacteria) [77] and ARDB (Antibiotic Resistance Genes Database) database [78]. The molecular and biological information on genes of pathogen-host interactions were predicted by PHI-base [79]. Carbohydrate-active enzymes were predicted by the Carbohydrate-Active enZYmes Database [80]. Type III secretion system effector proteins were detected by EffectiveT3 [81]. Default settings were used in all software unless otherwise noted.

### Pan-genome analysis

All complete genomic assemblies classified as *K. oxytoca* and *K. michiganensis* were downloaded from the NCBI database on 19 March 2019 with NCBI-Genome-Download scripts (https://github.com/kblin/ncbi-genome-download). Genomic assemblies of *K. pneumonia*, *K. quasipneumoniae*, *K. quasivariicola*, *K. aerogenes,* and *Klebsiella variicola* type strains also were manually obtained from the NCBI database. The quality of the genomic assemblies was evaluated by QUAST [82] and CheckM [83]. Genomes with N75 values of <10,000 bp, >500 undetermined bases per 100,000 bases, <90% completeness, and >5% contamination were discarded. The whole-genome GC content was calculated with QUAST [82]. All pairwise ANIm (ANI calculated by using a MUMmer3 implementation) values were calculated with the Python pyani package [34]. To avoid possible biases in the comparisons due to different annotation procedures, all the genomes were re-annotated using Prokka [84]. The pan-genome profile including core genes (99% < = strains <= 100%), soft core genes (95% < = strains < 99%), shell genes (15% < = strains < 95%) and cloud genes (0% < = strains < 15%) of 119 *Klebsiella* strains was inferred with Roary [85]. The generation of a 773,658 bp alignment of 858 single-copy core genes was performed with Roary [85]. The phylogenetic tree based on the presence and absence of accessory genes among *Klebsiella* genomes was constructed with FastTree [86] using the generalized time-reversible (GTR) models and the –slow, −boot 1000 option.

### Unique genes inference and analysis

Orthogroups of BD177 and 33 *Klebsiella* sp. (*K. michiganensis* and *K. oxytoca*) genome assemblies were inferred with OrthoFinder [36]. All protein sequences were compared using a DIAMOND [87] all-against-all search with an E-value cutoff of <1e-3. A core orthogroup is defined as an orthogroup present in 95% of the genomes. The single-copy core gene, pan gene families, and core genome families were extracted from the OrthoFinder output file. “Unique” genes are genes that are only present in one strain and were unassigned to a specific orthogroup. Annotation of BD177 unique genes was performed by scanning against a hidden Markov model (HMM) database of eggNOG profile HMMs [37]. KEGG pathway information of BD177 unique orthogroups was visualized in iPath3.0 [88].

## Supplementary Information

**Additional file 1 Figure S1.** Phylogenetic tree based on 16S rRNA gene sequences indicating the relationship between isolate BD177 with other type strains of the family Enterobacteriaceae, constructed using MUSCLE and MEGAX. (a) A maximum likelihood phylogenetic tree based on 16S rRNA gene sequences was assessed using bootstrap with 1000 replicates. *Pseudomonas aeruginosa* JCM5962 was selected as the outgroup. (b) A maximum parsimony phylogenetic tree based on 16S rRNA gene sequences was assessed using bootstrap with 1000 replicates.

**Additional file 2 Table S1.** Distance matrix of evolutionary divergence between 16 s rRNA gene sequences. This distance matrix was obtained by a bootstrap procedure (1000 replicates) and the Kimura 2-parameter model.

**Additional file 3 Table S2.** RefSeq assembly accession numbers and strain name of the 128 *Klebsiella sp.* genomes from the NCBI database used in this study.

**Additional file 4 Table S3.** Overview of the various metrics employed to evaluate strain BD177 and 128 *Klebsiella sp.* genomes using QUAST and CheckM.

**Additional file 5 Fig. S2.** Diagram of conserved genes per number of genomes from the pan-genome analysis by Roary.

**Additional file 6 Table S4.** ANI values (%) for each pairwise genome comparison of 119 genomes passed strict quality control.

**Additional file 7 Table S5.** General statistics about orthogroup sizes and the proportion of genes assigned to orthogroups by OrthoFinder.

**Additional file 8 Table S6.** Annotate results of the unique orthogroups of BD177 from comparative genomics by eggNOGmapper.

## Data Availability

The genome sequence data for the BD177 strain reported in this study can be retrieved from the National Center for Biotechnology Information (accession no. PRJNA602959). The other *Klebsiella sp.* strains genomes sequence analyzed in this study were downloaded from and are available in the GenBank database (https://www.ncbi.nlm.nih.gov/genbank/). The accession numbers of the 128 *Klebsiella sp.* genomes are shown in Additional file 3: Table S2.

## Abbreviations

SIT: Sterile insect technique
bp: Base pair
kb: Kilo base pairs
Mb: Mega base pairs
Gbp: Giga base pairs
PCR: Polymerase chain reaction
LB: Luria Bertani
tRNA: Transfer ribonucleic acid
rRNA: Ribosomal ribonucleic acid
sRNAs: Small ribonucleic acid regulators
N50: The sequence length of the shortest contig at 50% of the total genome length
N75: The sequence length of the shortest contig at 75% of the total genome length
GC content: The percentage of guanine (G) and cytosine (C) in a certain fragment of DNA or RNA or for an entire genome
ANI: Average Nucleotide Identity.
