# Supplementary figures and images for: Comparative genomics of Klebsiella michiganensis BD177 and related members of Klebsiella sp. reveal the symbiotic relationship with Bactrocera dorsalis
Source: BMC Genet. 2020 Dec 18;21(Suppl 2):138. doi: 10.1186/s12863-020-00945-0 (PMC7747454; doi:10.1186/s12863-020-00945-0)

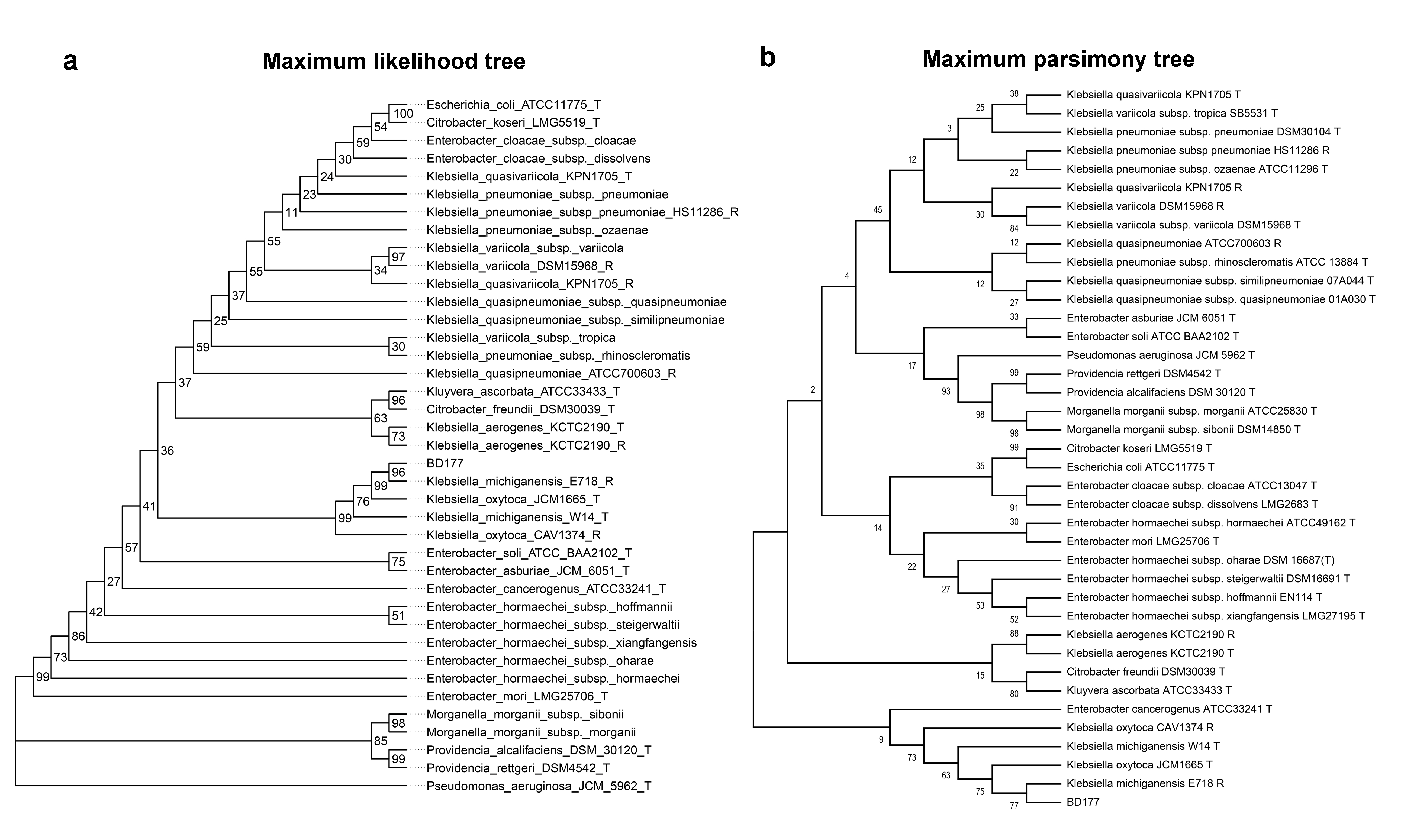

Supplement: Supplementary file 1 — Additional file 1 Figure S1. Phylogenetic tree based on 16S rRNA gene sequences indicating the relationship between isolate BD177 with other type strains of the family Enterobacteriaceae, constructed using MUSCLE and MEGAX. (a) A maximum likelihood phylogenetic tree based on 16S rRNA gene sequences was assessed using bootstrap with 1000 replicates. Pseudomonas aeruginosa JCM5962 was selected as the outgroup. (b) A maximum parsimony phylogenetic tree based on 16S rRNA gene sequences was assessed using bootstrap with 1000 replicates. [file 12863_2020_945_MOESM1_ESM.png]

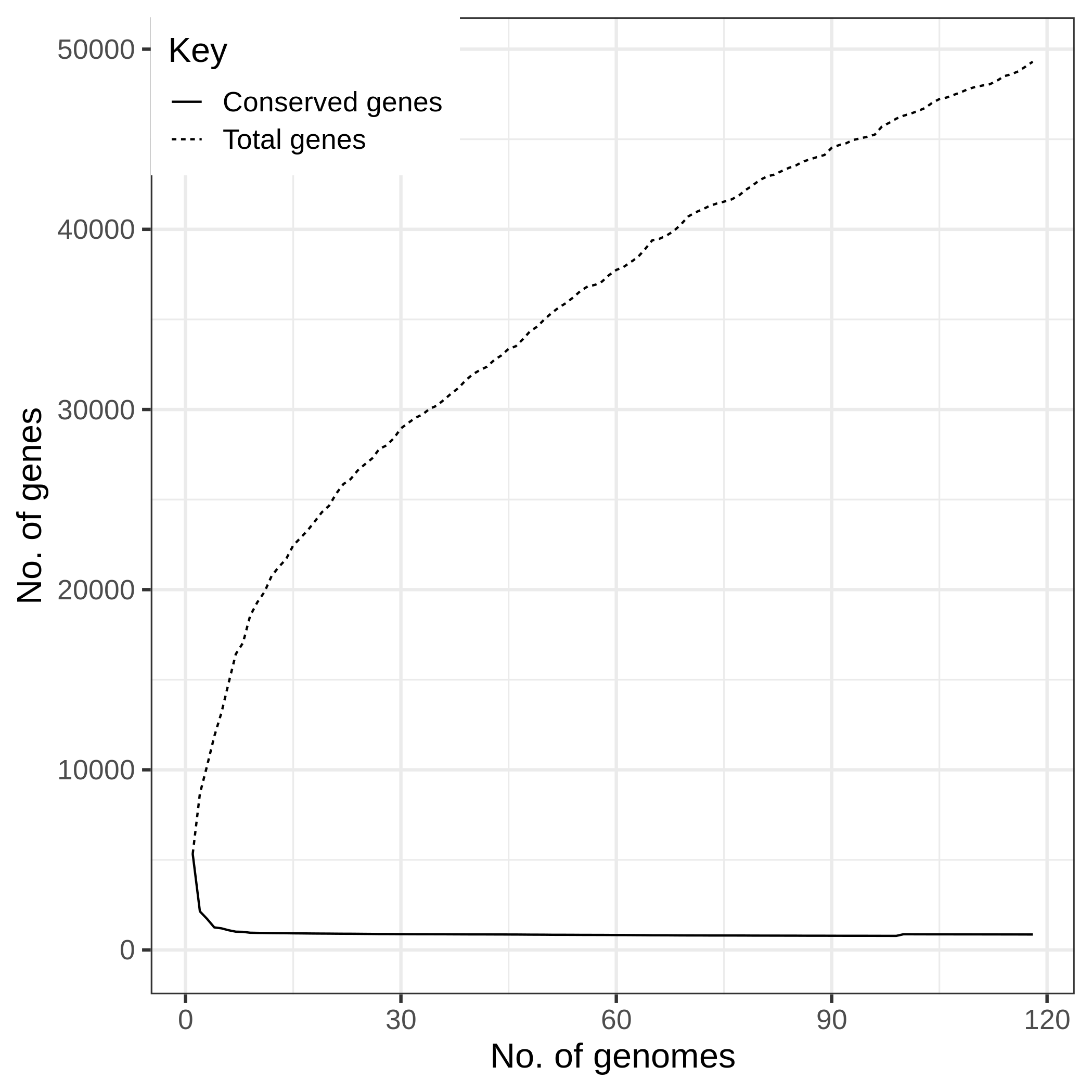

Supplement: Supplementary file 5 — Additional file 5 Fig. S2. Diagram of conserved genes per number of genomes from the pan-genome analysis by Roary. [file 12863_2020_945_MOESM5_ESM.png]
